# Demonstration of Three-Dimensional Indoor Visible Light Positioning with Multiple Photodiodes and Reinforcement Learning

**DOI:** 10.3390/s20226470

**Published:** 2020-11-12

**Authors:** Zhuo Zhang, Huayang Chen, Weikang Zeng, Xinlong Cao, Xuezhi Hong, Jiajia Chen

**Affiliations:** 1 Centre for Optical and Electromagnetic Research, South China Academy of Advanced Optoelectronics, South China Normal University, Guangzhou 510006, China; zhuo.zhang@coer-scnu.org (Z.Z.); huayang.chen@coer-scnu.org (H.C.); weikang.zeng@coer-scnu.org (W.Z.); xinlong.cao@coer-scnu.org (X.C.); 2 Department of Electrical Engineering, Chalmers University of Technology, Hörsalsvägen 9, 41296 Gothenburg, Sweden

**Keywords:** reinforcement learning, 3D indoor positioning, visible light positioning

## Abstract

To provide high-quality location-based services in the era of the Internet of Things, visible light positioning (VLP) is considered a promising technology for indoor positioning. In this paper, we study a multi-photodiodes (multi-PDs) three-dimensional (3D) indoor VLP system enhanced by reinforcement learning (RL), which can realize accurate positioning in the 3D space without any off-line training. The basic 3D positioning model is introduced, where without height information of the receiver, the initial height value is first estimated by exploring its relationship with the received signal strength (RSS), and then, the coordinates of the other two dimensions (i.e., X and Y in the horizontal plane) are calculated via trilateration based on the RSS. Two different RL processes, namely RL_1_ and RL_2_, are devised to form two methods that further improve horizontal and vertical positioning accuracy, respectively. A combination of RL_1_ and RL_2_ as the third proposed method enhances the overall 3D positioning accuracy. The positioning performance of the four presented 3D positioning methods, including the basic model without RL (i.e., Benchmark) and three RL based methods that run on top of the basic model, is evaluated experimentally. Experimental results verify that obviously higher 3D positioning accuracy is achieved by implementing any proposed RL based methods compared with the benchmark. The best performance is obtained when using the third RL based method that runs RL_2_ and RL_1_ sequentially. For the testbed that emulates a typical office environment with a height difference between the receiver and the transmitter ranging from 140 cm to 200 cm, an average 3D positioning error of 2.6 cm is reached by the best RL method, demonstrating at least 20% improvement compared to the basic model without performing RL.

## 1. Introduction

The developments of location-based mobile services and the Internet of Things urgently need stable and precise indoor positioning technologies [1]. As the widely deployed global positioning system (GPS) has poor coverage and accuracy in the indoor environment, indoor positioning systems (IPS) that employ alternative radio frequency (RF) technologies (e.g., Bluetooth, RFID, iBeacon, Wi-Fi, and near-field communication [2,3,4]) have been investigated. However, the RF-based IPS (e.g., 1–3/5–15/0.1–0.3 m with Bluetooth/Wi-Fi/UWB, respectively [5]) can be largely affected by electromagnetic interference and multipath effect in a congested environment [6]. Compared with RF technologies, the visible light positioning (VLP) system has features with immunity to electromagnetic interference and high tolerance to multipath interference thanks to the domination of the LOS signal [7,8,9,10,11]. By simultaneously providing illumination and positioning services with the existing indoor lighting equipment (e.g., light-emitting diode (LED)), VLP with high positioning accuracy (e.g., in the order of centimeters [7]) is considered as one low cost and high energy efficiency solution for localization in the indoor environment.

Comparing with the two-dimensional (2D) positioning on a horizontal plane at a known height, three-dimensional (3D) positioning using the same setup is more challenging. In such a case, one needs to map the received signal to one more dimension (i.e., height) which increases the search space for the positioning process. To find the correct position in a larger searching space, the positioning algorithm and/or the hardware in 3D VLP systems are more complex than the 2D ones. From the hardware perspective, 3D VLP systems based on either a single photodiode (PD) or multiple PDs have been proposed. In the single-PD system, 3D positioning has been achieved by combining information from both the PD and the other hardware either at the receiver (e.g., accelerometer [12], rotatable platform [13]) or at the transmitter (e.g., steerable laser [14,15]), which is not necessarily simpler than the multiple-PD system from the system complexity perspective. 3D positioning based on a low complexity receiver with one PD only has also been proposed, which has additional requirements for the radiation patterns or geometric arrangement of LEDs to avoid ambiguity in height estimation [16,17]. 3D VLP systems using multiple PDs have also been proposed, in which the spatial or angular diversity of PDs are explored to estimate the 3D position of the receiver [18,19,20]. Though it needs more PDs at the receiver, it does not have any special requirement for the transmitter [16,17] and has shown the potential to reduce the number of LEDs for a simpler transmitter [13].

For the 3D positioning algorithm, trilateration and triangulation based methods are widely employed, in which the geometric relationship between the receiver and light sources (e.g., distance [19], incidence/irradiance angles [18,20]) is estimated from the received signal. One popular way to evolve from a 2D VLP algorithm to a 3D one is to conduct a brute-force search on several parallel 2D layers at various heights. After obtaining the horizontal positions on all candidate layers at a pre-defined height set, the estimated 3D position is determined as the one that most likely fulfills the constraint among the coordinates of the three dimensions [16,21,22]. To improve the efficiency in height estimation, a fast search method based on the golden section search (GSS) algorithm has been proposed which can significantly reduce the running time [16]. To further improve the positioning accuracy, machine learning (ML) techniques with outstanding nonlinear fitting capability have been introduced to the VLP systems. Supervised learning (SL) based VLP systems (e.g., neural network [23,24,25], random forest [26], and *K*-nearest neighbor [27]) have been proposed. However, the SL based VLP systems require sufficient training data to be prepared in advance, which increases the system complexity [27]. The performance of the SL positioning algorithms is also largely affected by the quality of the training. To avoid the above drawbacks, training-data-free ML techniques, such as reinforcement learning (RL), have been employed. Previous studies show that the application of RL in 2D VLP offers high and robust positioning accuracy [28,29]. Though the RL based 2D positioning algorithm has shown a higher tolerance to the error of a priori height information than the conventional one, the height of the object is still assumed to be known in advance under the 2D VLP framework. Moreover, in many applications with mobile devices, the exact height of the receiver is often unknown and could vary dynamically in a range much larger than the height error tolerance of the RL based 2D VLP algorithm.

In this paper, a 3D VLP system using multiple PDs and reinforcement learning is proposed which realizes high accuracy for 3D positioning without needs of data for off-line training. In the 3D VLP system, we first make a coarse estimation of the receiver height by exploring its relationship with the received signal strength (RSS) and then calculate the other two coordinates in the horizontal plane using trilateration [30]. To achieve high 3D positioning accuracy, three methods based on RL with different height update strategies are proposed. Experiments are carried out to evaluate the performance of the proposed methods under different receiver sizes. The results show that when the height difference between the receiver and the transmitter is within [140, 200]-cm, compared with the case without machine learning (i.e., the Benchmark), all three proposed RL based methods can improve 3D positioning accuracy robustly. Unlike our previous 2D VLP work [28,29] that only estimates the position on a horizontal plane and still requires the height information as an input, this paper is an extension, include three new major contributions: (i) methods for 3D positioning are investigated that output coordinates in all three dimensions without a priori information about any dimensions; (ii) two novel reinforcement learning processes are devised specifically for 3D VLP, which target accuracy enhancement in the horizontal plane and the vertical dimension, respectively, and a combination of them offers the highest 3D positioning accuracy; (iii) the effectiveness of the proposed RL based 3D positioning methods are demonstrated experimentally.

The remainder of the paper is organized as follows. The operation principles of different 3D positioning methods, including the basic model and three RL based methods (i.e., Method 1/2/3) are explained in Section 2. Section 3 shows the experimental setup for 3D VLP, compares the performance of different positioning methods, and analyze the impact of the receiver size. Finally, Section 4 draws conclusions.

## 2. Operation Principle

**Figure 1:**
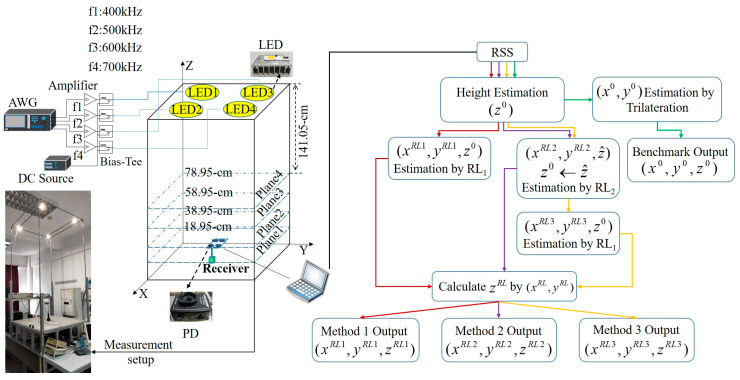
The 3D VLP system setup and the signal processing flow. The green, red, purple, and yellow lines represent the flow for the Benchmark, Method 1, Method 2, and Method 3, respectively. The inset shows the picture of our testbed.

A multi-PD VLP system with *M* (*M* ≥ 3) LEDs at the same height on the ceiling is considered in this study. Figure 1 shows the considered 3D VLP system setup and the signal processing flow, including the basic model without RL (later referred to as the benchmark), and three proposed methods that employ RL to improve positioning accuracy.

The *i-*th (*i* = 1, 2…*M*) LED is located at $\left(L_{i}^{x},L_{i}^{y},L_{}^{z}\right)$ and transmits a sinusoidal modulated signal with frequency *f_i_*. At the receiver, *N* PDs are facing up at the same height, and the *n-*th (*n* = 1, 2…*N*) PD is located at (*x_n_*, *y_n_*, *z*). The received signal of the *n*th PD from all the LEDs is represented by *s_n_*(*t*), whose power spectrum consists of *M* peak components at *f_i_* (*i* = 1, 2, …, *M*) [31]. After Fourier transformation, it can be expressed as [31]:

(1) $$S_{{}_{n}}^{f_{i}}=\frac{{\left(P_{i}\right)}^{2}{\left(m+1\right)}^{2}A^{2}\beta^{2}h^{2(m+m^{\prime })}}{4\pi^{2}dn,i^{2(}{}^{2+m+m^{\prime })}}$$

in which *P_i_* is the transmitted optical power of the *i*th LED, *A* is the PD area, *β* is the PD responsivity, *h* = $L^{z}-z$ is the height difference between the receiver and LEDs, *m* (*m’*) is the Lambertian radiation pattern order of the LED (PD) and *d_n_*,*_i_* is the distance between the *n*th PD and the *i*th LED. Note that the irradiance angle and incidence angle are assumed to be the same in (1) as the PDs (LEDs) are facing up (down). The RSS of these components obtained by the *N* PDs from the *M* LEDs can be represented by a vector.

(2) $$\mathrm{Rec}=\left[S_{1}^{f_{1}},\ldots ,S_{1}^{f_{M}},\ldots ,S_{{}_{N}}^{f_{1}},\ldots ,S_{{}_{N}}^{f_{M}}\right]$$

According to the location of the LEDs and PDs, we have:

(3) $${\left(x_{n}-L_{i}^{x}\right)}^{2}+{\left(y_{n}-L_{i}^{y}\right)}^{2}+h^{2}=d_{n,i}^{2}$$

### 2.1. Basic 3D Positioning Model

We first introduce a basic 3D positioning model, which is also referred to as benchmark later to show the accuracy improvement brought by the proposed reinforcement learning methods. Unlike the 2D VLP, the height of the receiver *z*, which equals to $L^{z}-h$, is unknown in the 3D VLP system and needs to be estimated. According to (1) and (3), the relationship between *h* and $S_{{}_{n}}^{f_{i}}$ can be written as:

(4) $$h\leq dn,i={\left[\frac{C}{S_{{}_{n}}^{f_{i}}}{\left(\frac{h}{dn,i}\right)}^{2(m+m^{\prime })}\right]}^{1/4}\leq {\left[\frac{C}{S_{{}_{n}}^{f_{i}}}\right]}^{1/4}$$

where $C=\frac{{\left(P_{i}\right)}^{2}{\left(m+1\right)}^{2}A^{2}\beta^{2}}{4\pi^{2}}$.

According to Equation (4), *h* is no more than the minimum value of *d_n_*_,*i*_. We denote a coarse estimation of *h* as *h_0_*, which equals *d_min_* (i.e., the minimum value of the rightest term in Equation (4) among all possible combinations of *N* PDs and *M* LEDs) and is expressed as:

(5) $$h0\;=\;d_{min}\;=\mathrm{mininum}\;{\left[\frac{C}{S_{{}_{n}}^{f_{i}}}\right]}^{1/4}\;\forall n\in [1,N],\forall i\in [1,M]$$

With *h_0_*, the 2D coordinates on the horizontal plane of the *N* PDs can be estimated by the conventional trilateration method using (1) and (3). Specifically, we estimate the *n*th PD’s 2D coordinates by solving the following equations:

(6) $$\left\{ \begin{array}{l} 2x_{n}(L_{b}^{x}-L_{a}^{x})+2y_{n}(L_{b}^{y}-L_{a}^{y})=d_{n,a}^{2}-d_{n,b}^{2}+{\left(L_{b}^{x}\right)}^{2}-{\left(L_{a}^{x}\right)}^{2}+{\left(L_{b}^{y}\right)}^{2}-{\left(L_{a}^{y}\right)}^{2}\\ 2x_{n}(L_{c}^{x}-L_{a}^{x})+2y_{n}(L_{c}^{y}-L_{a}^{x})=d_{n,a}^{2}-d_{n,c}^{2}+{\left(L_{c}^{x}\right)}^{2}-{\left(L_{a}^{x}\right)}^{2}+{\left(L_{c}^{y}\right)}^{2}-{\left(L_{a}^{y}\right)}^{2} \end{array}\right.$$

where *a*/*b*/*c* are the indexes of three different LEDs. As there are *M* LEDs on the ceiling, $C_{M}^{3}$ different pairs of equations can be established [30]. The output of trilateration is obtained by averaging these estimations to mitigate the impact of noise. Our positioning target is the coordinate of the center of the receiver. Assuming the PDs locate symmetrically at the corners of the receiver, the receiver position is obtained by averaging the estimated locations of the *N* PDs. The above 3D VLP system is referred to as the benchmark, whose output is (*x^0^*, *y^0^*, *z^0^* = *L^z^* − *h*_0_) (see Benchmark Output in Figure 1).

### 2.2. Reinforcement Learning To Enhance 3D Positioning Accuracy

*h_0_* derived from (5) is a coarse estimation of the actual height *h*. The difference between *h_0_* and *h* may not be minor, particularly when the PD is not right below any LED. Since the estimation of the other two coordinates requires the height information as an input, the coarse estimation of *h* causes error propagation in the benchmark, which results in low positioning accuracy in all three dimensions. Inspired by our previous study [29] that RL can offer high tolerance to inaccurate *h* in the 2D VLP system, we propose to use RL to improve the positioning accuracy of the 3D VLP system.

**Figure 2:**
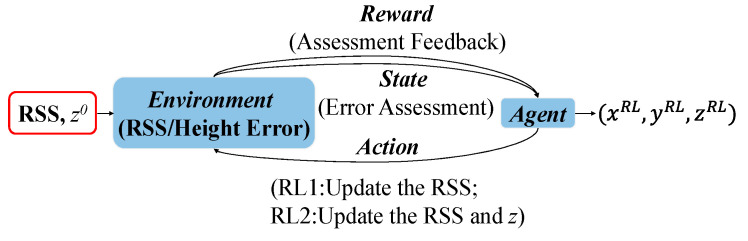
Schematic diagram of the reinforcement learning mechanism in the 3D VLP system.

The RL mechanism is shown in Figure 2, in which the *Agent* learns knowledge in the action-evaluation *Environment* and improves the *Action* by adapting to the *Environment* [32]. In the 3D VLP system, if the RSS and height are free from the impact of noise in RSS or height estimation error, we can get the exact 3D coordinates by using trilateration. Therefore, the *Environment* to be learned in the 3D VLP system is the error in RSS measurement and the height estimation (see the red box in Figure 2). In other words, the aim of RL is to learn and compensate for the above errors contained in the *Environment* to get a better estimation of the receiver position. As we have multiple PDs available at the receiver, the relative distances between PDs are fixed and can be used to assess the positioning error for reward calculation in RL. The relative distance error vector **E***_dis_* is used by the *Agent* to evaluate the *State* of *Environment*, which is defined as:

(7) $$E_{dis}=\{dis_{(}{{}_{i}}_{,}{{}_{j}}_{)}\;-d\hat{i}s_{(}{{}_{i}}_{,}{{}_{j}}_{)}\;|i\neq j;i,j=\;1,\;2..,N\}$$

**Figure 3:**
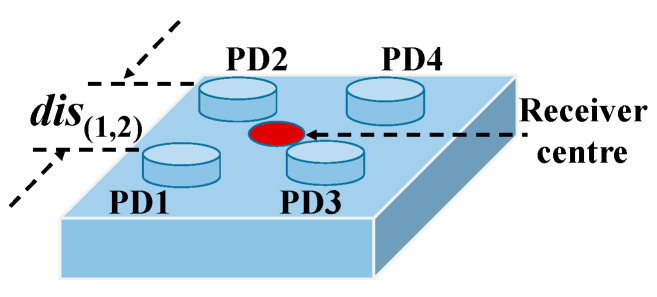
Receiver structure.

The $dis_{\left(i,j\right)}$($d\hat{i}s_{\left(i.j\right)}$) in Equation (7) denotes the real (calculated) distance between the *i-*th and *j-*th PDs. The *dis_(1, 2)_* of a four-PD receiver is shown in Figure 3 as an example. The *State* and *Reward* in the interaction between the *Agent* and *Environment* are defined as the maximum and average value of **E***_dis_*, respectively:

(8) $$State=\left\{ \begin{array}{l} i,\mathrm{if}\;\alpha_{i-1}\;<\max(E_{dis})\leq \alpha_{i}\;\mathrm{for}\;1\leq i<G\\ G,\mathrm{if}\max(E_{dis})\geq \alpha_{G-1} \end{array}\right.,$$

(9) $$Reward=\left\{ \begin{array}{l} \frac{K-i}{K-1}*100,\;\mathrm{if}\;r_{i-1}<\mathrm{average}(E_{dis})\leq r_{i}\;\mathrm{for}\;1\leq i<K\\ 0,\mathrm{if}\;\mathrm{average}\left(E_{dis}\right)\geq r_{K-1} \end{array}\right.,$$

where (*α*_0_, *α*_1_, …, *α_G-1_*) and (*r*_0_, *r*_1_, …, *r_K-1_*) are pre-determined constants based on accuracy requirements, *G* and *K* are the numbers of possible values for the *State* and *Reward*, respectively. The learning process in RL uses an action-evaluation strategy, where the consequences of actions (i.e., *Reward*) is used as the metric to help find the optimal action at a certain *State* of *Environment*. If the current *State* is not the target state (e.g., 1 in our study), the *Agent* takes an action to adjust the RSS and height coordinate.

There are different ways to conduct 3D positioning incorporating the RL. Pseudocode 1 shows the pseudocode for two methods with different height update strategies, namely RL_1_ and RL_2_. The RL_1_ is used in Method 1 that adjusts the RSS without changing *h* except for the last action in learning (i.e., *h* is fixed to be *h_0_* when adjusting the RSS and only gets updated after the final RSS is obtained), while the RL_2_ is used in Method 2 that adjusts RSS and *h* sequentially in each action. Specifically, in Method 2, $\left(x_{n}^{new},y_{n}^{new}\right)$ is obtained by using the updated RSS and ${\hat{d}}_{n,i}^{}={\left(Ch^{2(m+m^{\prime })}/S_{{}_{n}}^{f_{i}}\right)}^{\frac{1}{2(2+m+m^{\prime })}}$ based on trilateration in Equation (3), and then height difference is updated as $\hat{h}$ by averaging the *N* height differences between each LED and the receiver’s plane, which can be expressed as:

(10) $$\hat{h}=\frac{1}{N}\sum_{n=1}^{N}\sqrt{{\left(Ch^{2(m+m^{\prime })}/S_{{}_{n}}^{f_{i}}\right)}^{\frac{1}{2+m+m^{\prime }}}-{\left(x_{n}^{\mathrm{new}}-L_{i}^{x}\right)}^{2}-{\left(y_{n}^{\mathrm{new}}-L_{i}^{y}\right)}^{2}}$$

**Pseudocode 1:** Pseudocode for Method 1 and Method 2
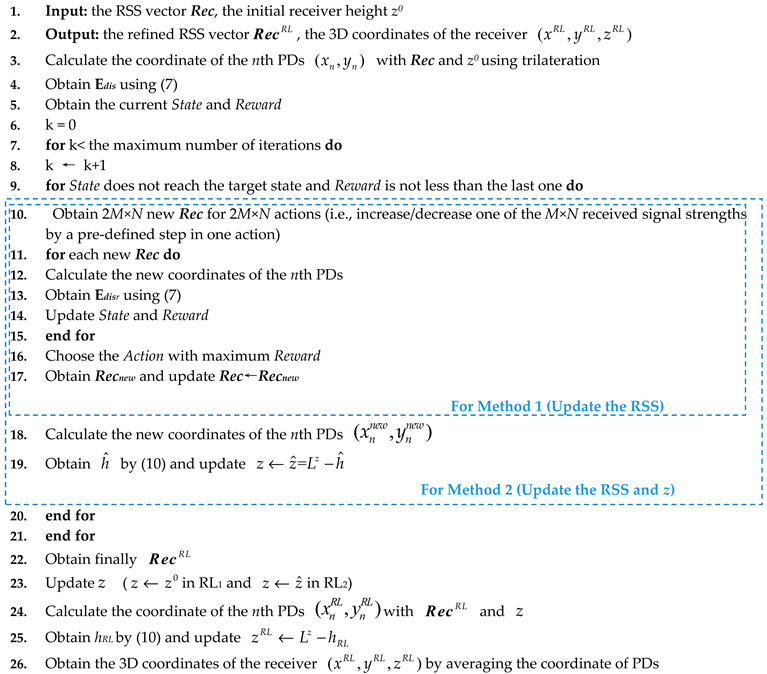
* $z^{RL}$ corresponds to $z^{RL1}$ and $z^{RL2}$ for Method 1 and Method 2, respectively.

For the RSS adjustment in the RL_1_/RL_2_, each time one element of the RSS vector ***Rec*** is increased or decreased by step which is a minimum step to adjust the RSS values. After taking an action that modifies ***Rec*** (in RL_1_ or RL_2_) and *h* (in RL_2_), the 3D coordinates of all PDs are obtained via trilateration and used to calculate its *Reward* based on a new **E***_dis_* according to Equation (9). The *Agent* chooses the *Action* with the maximum *Reward*, and update the *State* according to Equation (8).

Both methods continue the learning process until the target state or the maximum number of iterations. After learning, the estimated 3D coordinates of PDs after the last action in RL are saved. The receiver’s 3D coordinates (i.e., (*x^RL1^*, *y^RL1^*^,^ *z^RL1^*=*L^z^*-*h_RL1_*) in Method 1 and (*x^RL2^*, *y^RL2^*^,^ *z^RL2^*=*L^z^*-*h_RL2_*) in Method 2) are obtained by averaging the coordinates of PDs and used as the final outputs (see Method 1 Output and Method 2 Output in Figure 1).

It is worth noting that the two methods concentrate on positioning accuracy improvement in the horizontal plane and height, respectively. In the RL_1_, the learning process only puts the efforts to optimize the X and Y coordinates in the horizontal plane, while the RL_2_ does one-step refinement for both the height and RSS in each action. It is also shown in the results (see Section 3), the two methods cannot achieve positioning accuracy improvement in all three dimensions simultaneously. Therefore, we combine the RL_1_ and the RL_2_, which is referred to as Method 3. Since our previous research in [29] shows that reinforcement learning can tolerate the inaccuracy of *h* to some extent, in Method 3 we use the RL_2_ to update *h* and RSS, which are followed by the RL_1_ to update the X and Y coordinates. Finally, the height estimation is refined according to Equation (10), and (*x^RL3^*, *y^RL3^*, *z^RL3^*=*L^z^*^-^*h_RL3_*) is obtained (see Method 3 Output in Figure 1). The pseudocode for Method 3 is shown in Pseudocode 2.
**Pseudocode 2:** Pseudocode for Method 3**1.** Input: the RSS vector *Rec***2.** Output: Coordinate of the receiver $\left(x^{RL3},y^{RL3},z^{RL3}\right)$. **3.** Estimate *h_0_* with (5) and *z^0^*=$L^{z}-$*h*_0_**4.** Run *RL_2_* to obtain *Rec^RL^* and $\hat{z}$
**5.** Update $Rec\leftarrow Rec^{RL}$,$z^{0}\leftarrow \hat{z}$**6.** Run *RL_1_* to obtain the 3D coordinate of the receiver $\left(x^{RL3},y^{RL3},z^{0}\right)$
**7.** Refine height *z^RL3^***8.** Obtain the final 3D coordinates of the receiver $\left(x^{RL3},y^{RL3},z^{RL3}\right)$

To better illustrate the RL processes in different 3D VLP methods, Table 1 summarizes the features of the three proposed methods. The RL-based 2D VLP method (i.e., PWRL in [29]) is also listed for comparison.

## 3. Experiment Investigation

### 3.1. Experimental Setup

**Figure 4:**
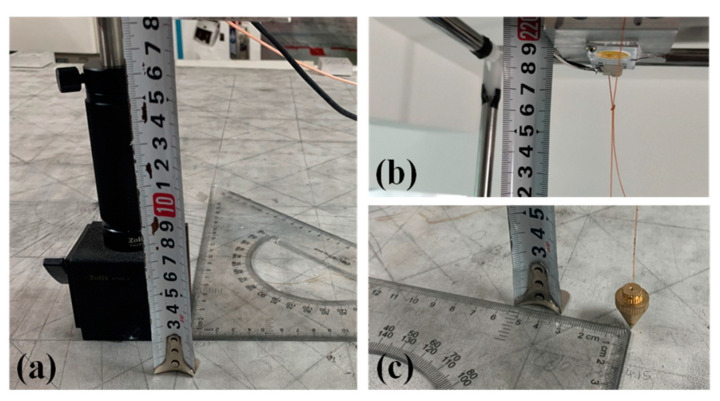
Measurements of the ground truth locations of (**a**) PD, (**b**) and (**c**) LED.

The performance of the proposed 3D VLP methods is investigated experimentally. Figure 1 shows the experimental setup. There are four LEDs (Cree CXA2435) on the ceiling with coordinates of (24.2, 19.8, 218.9), (83.5, 19.7, 218.9), (22.7, 78.1, 218.9), (82.6, 77.8, 218.9) in centimeter (cm), respectively. Four sinusoidal signals of frequency (400/500/600/700 kHz) from four signal generators are amplified and then combined with direct current (DC) signals via Bias-Tees (ZFBT-4R2GW+) to drive the four LEDs, respectively. As shown in Figure 3, the receiver consists of four PDs (PDA100A2) on the four corners. To ensure that the signal from all four LEDs can be received by the PD (field of view: ~60°) in the 120 cm × 120 cm area, the height difference between the PD and the LED of our test space should be larger than 71 cm. To investigate the impact of receiver size on the performance of the proposed 3D VLP methods, the distance between adjacent PDs is adjusted (i.e., *dis_(1,2)_* = 10/20/30/40 cm). In order to get ground truth locations of PDs and LEDs, we divide the area of a solid aluminium plate into many 10 cm × 10 cm grids with a ruler/tape measure which has the resolution of 1 mm and use the lower left side as the origin. The PD is mounted with an optical mounting post on a base which is moved on the grid to change the 2D coordinates on horizontal planes (see Figure 4a). The height of the PD is adjusted by changing the length of the optical mounting post on the base, and is measured manually with a ruler. The horizontal and height coordinates of LEDs are determined by finding their projections on the solid aluminium plate and their distance to this plate with the help of a plumb bob (see Figure 4b,c). To lower the measurement error, the averaged value of multiple measurements is used as ground truth locations. We take measurements at four test planes of different heights with 20 cm spacing, whose Z coordinates are 18.95/38.95/58.95/78.95 cm, corresponding to 199.95/ 179.95/159.95/139.95 cm for *h*, respectively. The height difference between the receiver and the ceiling in the testbed is about [140, 200]-cm, which emulates the cases of positioning a hand-held device in a typical office environment. Note that the tilt of a hand-held device could severely affect the positioning accuracy as Equation (1) no longer holds. As the average elbow height for a mixed male/female human population is 104.14 cm when he/she stands up [33], this offers about ± 30 cm margin for a room with a ceiling height of 270 cm. In case the height difference is larger, a stronger light source is needed to guarantee a reasonable signal-to-noise ratio (SNR) for high accuracy positioning [30]. For each test plane, four PDs are adjusted to the same height, and samples are taken at 49 uniformly distributed locations in the 120 cm × 120 cm area. The RSS at the receiver is measured using a spectrum analyzer (8593E, Agilent, Elgin, IL, USA) with a sweep time of 30 ms and averaged over 10 measurements. For example, the measured RSS in the center of Plane 4 are 0.354-μW, 0.292-μW, 0.309-μW, 0.319-μW for the sine wave signals from the four LEDs, respectively. For a practical receiver of small form factor, discrete Fourier transform of the temporal samples from an analog-to-digital converter can be conducted to measure the signal strength at different frequencies. The detailed parameters of the experimental setup are listed in Table 2.

To balance running time and positioning accuracy, we set ${}_{▵}$*step* to 0.1 μw to adjust ***Rec*** in each action during the learning process. *K* = *G* = 5, (*α*_0_, *α*_1_, *α*_2_, *α*_3_, *α_4_*) = (0, 0.2, 0.5, 1, 2) in cm, and (*r*_0_, *r*_1_, *r*_2_, *r*_3_*, r_4_*) = (0, 0.05, 0.125, 0.25, 0.5) in cm. In general, the accuracy performance is improved when the number of iterations increases and exhibits a trend of convergence when the number of iterations exceeds a certain value. The number of iterations shall not be too small to achieve the state of convergence. On the other hand, since the processing time and computational complexity of the algorithm increase with a larger number of iterations, the number of iterations shall not be too large. Therefore, the maximum allowable number of iterations in RL based methods is set to 1000 empirically in this experiment to balance the complexity and positioning accuracy.

### 3.2. Performance Evaluation

**Figure 5:**
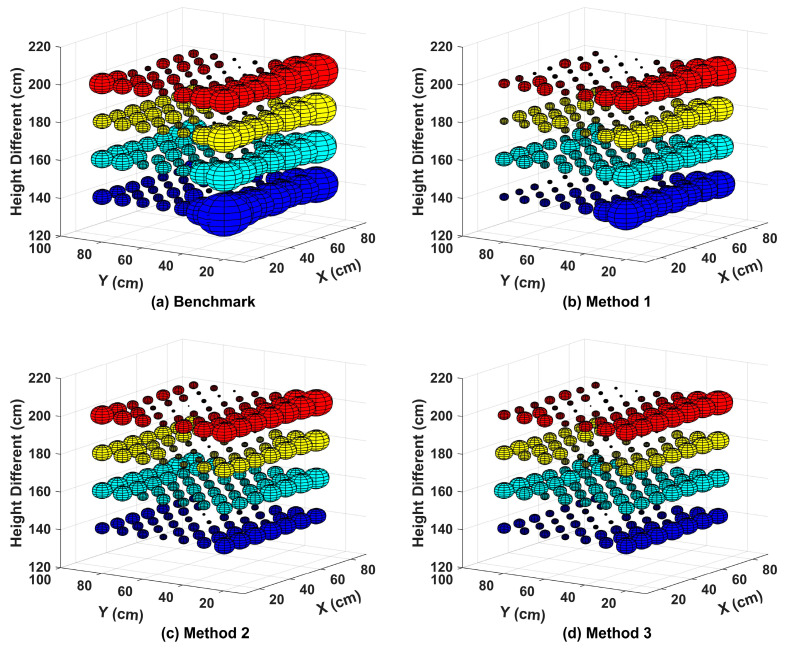
Spatial distribution of the 3D positioning error at different heights in the case of dis_(1,2)_ = 40 cm for the (**a**) Benchmark, (**b**) Method 1, (**c**) Method 2, and (**d**) Method 3.

We run Method 1/2/3 off-line with MatLab (MathWorks, Natick, MA, USA) on a desktop computer (i5 processor @2.29 GHz (Intel, Santa Clara, CA, USA) with 16 GB RAM) and the measured average processing time is 0.96/0.44/0.69-s, respectively. Figure 5 shows the spatial distribution of 3D positioning error for four different positioning methods (i.e., the benchmark and methods 1/2/3) when dis_(1,2)_ equals to 40 cm. The 3D/2D positioning errors are the Euclidean distance between the real coordinates and the calculated coordinates of the receiver in the 3D/2D space, respectively. To illustrate the 3D positioning accuracy intuitively, we take the actual position of the sampling point as the center of the sphere and the 3D positioning error as the radius of the sphere. The radius $r_{sphere}$ is defined as:

(11) $$r_{sphere}=\sqrt{{\left(x-x_{real}\right)}^{2}+{\left(y-y_{real}\right)}^{2}+{\left({z-z}_{real}\right)}^{2}},$$

where (x,y,z) denotes the output of the positioning algorithms and $\left(x_{real},y_{real},z_{real}\right)$ is the real coordinate of the receiver. The non-uniform distribution of errors is observed, which is the interplay of there location-dependent factors: (a) SNR which is higher at the center of test plane, (b) inaccurate a priori information about the VLP system (e.g., m and m’ in (1)) that may cause significant overestimation or underestimation of the distance between PD and LED, and (c) the error in approximating the actual height difference with *h*_0_ in Equation (5) which varies for different incidence/irradiance angles of the PD-LED pair used in the calculation of *h*_0_. 

At the edges of test planes where the SNR is lower, the 3D positioning error is larger than that in the central of test planes with higher SNR. If the overestimation of the distance between the PD and LED happens (e.g., a result due to factor (b)), the approximation error in Equation (5) will be larger. For example, we find that the Lambertian model for LED1/LED2 with the parameters in Table 2 causes overestimation of the distance between LED and PD. This leads to significant larger positioning errors in the region with smaller Y which uses LED1/LED2 to calculate *h*_0_. In general, all three RL based methods achieve higher 3D positioning accuracy than the benchmark in the test planes. Method 2 can reduce the error of some points to very small (e.g., test points on the left half of Figure 5c). However, the positioning error with Method 1 is more uniformly distributed in some planes (e.g., *h* = 139.95/159.95 cm in Figure 5b,c). Regardless of the height of the receiver’s plane, Method 3 offers the best performance among the four methods.

**Figure 6:**
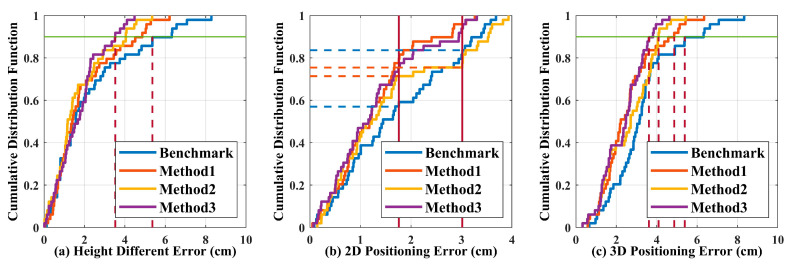
The cumulative distribution function of (**a**) height, (**b**) 2D, and (**c**) 3D positioning errors at Plane 2 in the case of dis_(1,2)_ = 40 cm.

To further analyze the impact of RL on positioning errors in different dimensions, Figure 6a–c give the cumulative distribution function (CDF) of height/2D/3D error, respectively. Here, 2D error represents the error in the horizontal plane. Plane 2 (i.e., h = 179.95 cm) is used as an example in Figure 6. As shown in Figure 6a–c, all three RL based methods can reduce the height/3D positioning error. For the height dimension, the improvement in Method 3 is most significant, which can reduce the height error from ~5.4 cm to ~3.5 cm for 90% of the test points. Thanks to the additional height update procedure, Method 2 outperforms Method 1 in terms of height estimation accuracy. As shown in Figure 6b, Methods 1 and 3 perform similarly and reduce the 2D positioning error significantly when compared with the Benchmark. For Method 2, though more points are having lower positioning error when compared with the Benchmark, the number of points with larger positioning error also increases. For example, the ratios of points with 2D positioning error of ≤1.76 cm (≥3.0 cm) are 71% and 57% (25% and 17%) for Method 2 and the Benchmark, respectively. This is consistent with the enhanced non-uniformity by Method 2 shown in Figure 5c. In Figure 6c, the 3D positioning error of 90% test points with the Benchmark is less than ~5.4 cm, which can be reduced to less than ~4.9 cm, ~4.0 cm, and ~3.6 cm by Methods 1–3, respectively. As Method 3 exhibits superior performance in both the height dimension and the XY plane, it offers the best 3D positioning performance among the four tested algorithms.

Figure 6 implies that Method 2 outperforms Method 1 in the height dimension, while Method 1 outperforms Method 2 in the horizontal plane. Method 3 inherits the advantages of Method 1 and Method 2, performing the best in all dimensions. The performance superiority of the different RL based methods at different dimensions can be attributed to their unique learning mechanisms (see Figure 2 and Pseudocodes 1 and 2). The RL_1_ focuses on optimization of the 2D positioning error, and updates the height estimation only at the end of the learning process, while the RL_2_ updates the height estimation in each action, which improves the height estimation accuracy but no further optimization in the horizontal plane. For Method 3, it first uses RL_2_ to get a better estimation of the height and then uses RL_1_ to optimize the rest two coordinates (see Pseudocode 2). For the CDF, the tested error is a continuous random variable. As we keep each measured point as an individual test, there are always some steps in the CDF curves. As shown in Figure 6, we always give the upper bound of test errors for the proposed RL based algorithms (i.e., Methods 1–3) but the lower bound of test errors for the Benchmark. It is a conservative way to show the benefits brought by the RL. More test points might help to estimate more accurate improvement but would not make the concluding results not true.

**Figure 7:**
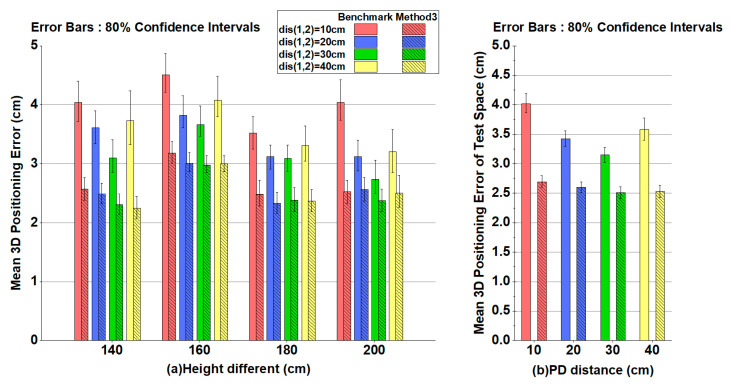
(**a**) Mean 3D positioning error at different heights for dis_(1,2)_ = 10/20/30/40 cm with Benchmark/Method 3. (**b**) Mean 3D positioning error in the test space for dis_(1,2)_ = 10/20/30/40 cm with Benchmark/Method 3.

Figure 7a shows the mean 3D positioning error obtained by the Benchmark and Method 3 for *dis*_(1,2)_ = 10/20/30/40 cm at different heights. 80% confidence intervals of the positioning error are also given in Figure 7a (i.e., the vertical bars). The improvement of 3D positioning accuracy with RL is obvious. The upper bounds of Method 3 are even smaller than the lower bounds of the Benchmark. Under different distances between adjacent PDs, Method 3 obtains a mean 3D positioning error below 3.2 cm regardless of the size of the receiver. The results also indicate that the performance of the two methods varies randomly in small ranges with respect to the height of test plane. Figure 7b shows the mean 3D positioning error obtained by the Benchmark and Method 3 for *dis*_(1,2)_ = 10/20/30/40 cm in the entire test space. The average 3D positioning errors with different receiver sizes are within [2.51, 2.69] cm and [3.15, 4.02] cm for Method 3 and the Benchmark, respectively, revealing an obvious reduction of the average 3D positioning error by at least 20%. Moreover, it also clearly indicates that the positioning performance is more stable (i.e., less variation of positioning errors) when the RL is implemented.

## 4. Conclusions

A 3D indoor VLP system with reinforcement learning to enhance the positioning accuracy is proposed and experimentally investigated. The three proposed RL based methods share the *Agent-Environment* interaction framework with properly defined *State/Action/Reward*, but employ different height update strategies. The experimental results show that thanks to the learning process, all three RL based positioning methods outperform the Benchmark in terms of 3D positioning accuracy. The results also verify that Method 1 (Method 2) with RL_1_ (RL_2_) offers a significant improvement in the horizontal plane (height dimension) over the Benchmark. By combining RL_1_ and RL_2_, Method 3 offers the highest positioning accuracy not only in the 3D space but also in the height dimension and the horizontal plane, respectively. For the test planes with height difference from 140 cm to 200 cm, the mean 3D positioning error has been significantly improved (>20%) by Method 3 compared with the Benchmark. Moreover, the RL also reduces the variation of the 3D position error compared to the Benchmark with receivers of different sizes.

## Figures and Tables

**Table 1:** Summary of different RL-based VLP methods.

Method RL Element	3D VLP	3D VLP	3D VLP	2D VLP
	Method 1	Method 2	Method 3	PWRL [29]
*Input*	(1) Measured RSS and (2) height estimated based on the basic 3D positioning model			(1) Measured RSS and (2) exact height
*Environment*	Errors in RSS measurement and height estimation			RSS error
*Action*	RSS adjustment under an estimated height(RL_1_)	RSS and height adjustments (RL_2_)	RSS adjustment (in both RL_1_ and RL_2_) and height adjustment (in RL_2_)	Only RSS adjustment, where height is known.
*State*	Determined by the relative distance error with (8)			
*Reward*	Determined by the relative distance error with (9)			

**Table 2:** Experimental parameters.

Parameter	Value
Space size(length × width × height)	120 × 120 × 220 (cm)
Coordinates of LED1/LED2/LED3/LED4	(24.2, 19.8, 218.9)/
	(83.5, 19.7, 218.9)/
	(22.7, 78.1, 218.9)/
	(82.6, 77.8, 218.9) (cm)
*f1/f2/f3/f4*	400/500/600/700 (kHz)
LED voltage	18.0 (V)
LED current	0.32 (A)
Lambertian order of LED (*m*)	1.78
Lambertian order of PD (*m*’)	3.56
Distance between PD1 and PD2 (*dis_(1,2)_*)	10/20/30/40 cm
Heights of Plane 1/2/3/4	18.95/38.95/58.95/78.95 (cm)
Height difference between receiver 1/2/3/4 to LEDs (*h*)	199.95/179.95/159.95/139.95 (cm)
